# Generalized hybrid coronary revascularization vs. conventional off-pump coronary artery bypass grafting for multivessel coronary artery disease

**DOI:** 10.3389/fcvm.2025.1459072

**Published:** 2025-02-21

**Authors:** Shuai Zhang, Ping Li, Guang Li, Yunfeng Yan, Tao Sun, Ji Lin, Chenhao Zhang, Shuo Liu, Zheng Qu, Bin You

**Affiliations:** ^1^ Beijing Anzhen Hospital, Capital Medical University, Beijing, China; ^2^ Beijing Institute of Heart Lung and Blood Vessel Diseases, Beijing, China

**Keywords:** generalized hybrid coronary revascularization, nonclassical hybrid coronary revascularization, off-pump coronary artery bypass grafting, minimally invasive cardiac surgery, multivessel coronary artery disease

## Abstract

**Background:**

Hybrid coronary revascularization (HCR) has been demonstrated as a safe and effective revascularization strategy in selected patients with multivessel coronary artery disease; however, the inclusion criteria are too strict.

**Objectives:**

This study was conducted to compare in-hospital and midterm outcomes after generalized HCR and off-pump coronary artery bypass (OPCABG) in patients with multivessel coronary artery disease.

**Methods:**

We proposed a generalized idea of HCR. First, the PCI for non-LAD vessels suitable for coronary stents was performed. Then, MICS-CABG for LIMA to the LAD and saphenous to other non-LAD vessels that were not suitable for stents or stenting failed. Propensity score matching was used, and 222 patients (*n* = 111 in both the generalized HCR and OPCABG groups) were enrolled in the study. The primary endpoint was a major adverse cardiac or cerebrovascular event (MACCE) over midterm follow-up, and the secondary endpoints were in-hospital outcomes.

**Results:**

No signiﬁcant difference was observed in the cumulative rate of MACCE (9.9% vs. 16.2%; HR, 0.567; 95% CL, 0.268–1.201; *P* = 0.138) between the generalized HCR and OPCABG groups. The residual SYNTAX score was similar between two groups (6.3 ± 5.5 for generalized HCR vs. 6.8 ± 5.3 for OPCABG; *P* = 0.486). Compared with OPCABG, generalized HCR was associated with a signiﬁcantly lower intra-aortic balloon pump (IABP) implantation rate (2.7% vs. 9.9%; *P* = 0.027) and shorter postoperative length of stay (6.3 ± 3.2 vs. 7.7 ± 3.0; *P* = 0.001).

**Conclusions:**

The generalized HCR procedure appears to be safe and efﬁcacious, with outcomes similar to those of standard off-pump CABG and satisfactory completeness of revascularization.

## Introduction

Conventional coronary artery bypass grafting (CABG) is still the gold standard treatment strategy for patients with multivessel coronary artery disease. A signiﬁcant number of patients will beneﬁt from CABG because of the favourable long-term patency rate (>90% at 10 years) of the left internal mammary artery (LIMA) to the left anterior descending coronary artery (LAD) graft (1). However, conventional open-chest CABG is an invasive and high-risk procedure for patients with uncontrolled diabetes or multiple comorbidities, which easily results in sternal wound nonhealing and infection (2, 3).

Minimal-access coronary revascularization, represented by minimally invasive direct coronary artery bypass grafting (MIDCAB), can effectively reduce perioperative trauma and yield satisfactory long-term results (4). Hybrid coronary revascularization (HCR), which combines the procedure of MIDCAB to LAD and percutaneous coronary intervention (PCI) for non-LAD lesions, has been demonstrated to be a very attractive alternative for coronary revascularization in selected patients with multivessel lesions (5). Many studies have reported similar major adverse cardiac and cerebrovascular events (MACCEs) between HCR and conventional CABG (6–9). Generally, HCR has strict inclusion criteria: LAD lesions are suitable for LIMA grafting, and non-LAD lesions are suitable for PCI stenting. Patients with non-LAD lesions not appropriate for stents must return to conventional CABG and cannot benefit from HCR.

On the foundation of having a good command of minimally invasive cardiac surgery-CABG (MICS-CABG) for multivessel coronary artery disease, we proposed the generalized idea of a hybrid coronary revascularization strategy. The PCI for non-LAD vessels suitable for coronary stents was performed immediately after the coronary angiography assessment. Then, anastomosis was performed via MICS-CABG, including LIMA to the LAD artery and saphenous to other non-LAD vessels that were not suitable for coronary stents or for which stenting failed. This study was conducted to objectively evaluate the safety and efficiency of this HCR strategy in patients with multivessel coronary artery disease.

## Methods

### Definitions of some critical abbreviations used in this article

MIDCAB, minimally invasive direct coronary artery bypass grafting for single-vessel LIMA-LAD.

MICS-CABG, minimally invasive cardiac surgery for multivessel bypass grafting.

CABG, coronary artery bypass grafting with median thoracotomy.

Traditional HCR, Hybrid coronary revascularization involving PCI and the MIDCAB procedures.

Nonclassical HCR, hybrid coronary revascularization involving the PCI and MICS-CABG procedures.

Generalized HCR, included traditional HCR and nonclassical HCR.

### Patient selection

Inclusion criteria for generalized HCR strategy:

(1) Unsuitable LAD lesion for PCI (i.e., chronic total occlusion, excessive tortuosity, severe diffuse lesion). At least one non-LAD vessel is suitable for PCI.
(2) High-risk or relative contraindications to median sternotomy (i.e., malignancies, severe obesity, uncontrolled diabetes, chronic obstructive pulmonary disease, chronic renal failure, end-stage arterial occlusive disease, history of stroke with paraplegia) (10).
(3) Patient choice, patients who want to avoid median sternotomy may choose HCR.

Exclusion criteria for the generalized HCR strategy:

(1) Congestive heart failure with haemodynamic instability (especially when the ultrasonically assessed ejection fraction was less than 35%).
(2) Need for a concomitant operation (valve repair or replacement).
(3) Left subclavian artery and LIMA stenosis.
(4) Contraindications for anticoagulation therapy (high haemorrhagic tendency).

First, the interventional cardiologists performed the angiographic evaluation. Patients who met the inclusion criteria mentioned above were identified. The surgical team and interventional team discussed and decided whether to perform HCR. The implementation of any treatment strategy required the consent of the patients. Coronary stents can be performed at the same stage to avoid the side effects of repeated coronary angiography. Of course, staged PCI can also be performed for multi-vessel or more complex lesions.

**Figure 1:**
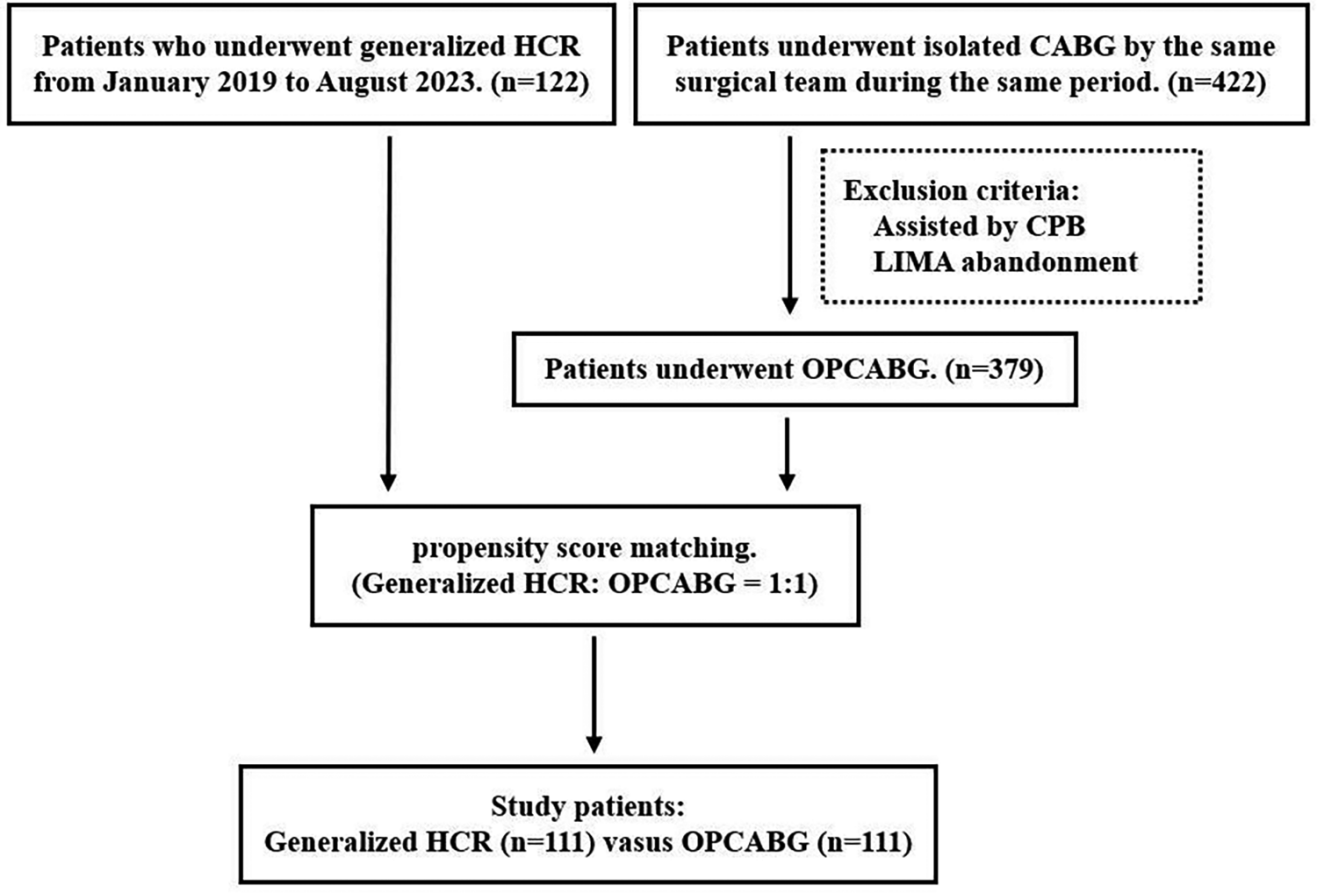
Flow chart of patient selection. HCR, hybrid coronary revascularization; CABG, coronary artery bypass grafting; CPB, cardiopulmonary bypass; LIMA, left internal mammary artery; OPCABG, off-pump coronary artery bypass grafting.

From January 2019 to August 2023, 122 patients underwent generalized HCR at the Minimally Invasive Cardiac Surgery Center of Beijing Anzhen Hospital. During the same period, 422 patients underwent isolated CABG through median sternotomy performed by the same surgical team. After screening these patients with the exclusion criteria, 379 patients were retained. Using propensity score matching, a total of 222 patients, 111 of whom were in either of the 2 groups, were ultimately enrolled in the study (Figure 1). The matching criteria included demographic information, comorbidities, and coronary anatomy variables known to be risk factors for revascularization. This study was approved by the Institutional Review Board of Anzhen Hospital.

## Surgical procedure

### Generalized HCR

After the coronary angiography evaluation, the interventional cardiologists performed PCI with drug-eluting stents (DESs) for non-LAD vessels, which were suitable for coronary stents. It is important to note that we do not require all the non-LAD vessels to be suitable for stents. We can address the rest of the non-LAD lesions in the following MICS-CABG procedure. One month after PCI, anastomosis was performed with the MICS-CABG, which included LIMA to the LAD and saphenous to other non-LAD vessels that were not suitable for coronary stents. 6–12 cm left anterolateral muscle-sparing mini-thoracotomy was performed in the fourth or ﬁfth intercostal space. One-lung ventilation was used to facilitate exposure. After the LIMA was harvested, distal anastomosis of the *in situ* LIMA-LAD graft was completed with the aid of a stabilizing device. The proximal saphenous vein was anastomosed to the ascending aorta with the help of a partial occluding clamp, and the distal part was sequentially anastomosed to the non-LAD vessels. All the grafts were tested with a coronary flow meter to confirm patency. All the MICS-CABG procedures were performed without the use of cardiopulmonary bypass. The procedure used for MIDCAB was the same as that used for single LIMA-LAD bypass.

For the perioperative anticoagulation strategy, a loading dose of 300 mg of aspirin plus 300 mg of clopidogrel was administered before the PCI procedure. The aspirin dosage was 100 mg/day, while clopidogrel was administered as a maintenance dose of 75 mg/day for 1 month after coronary intervention. When the patients were readmitted to the hospital, P2Y12 inhibitors were stopped for at least 5 days before MICS-CABG, while 100 mg/day aspirin was continued perioperatively. For postoperative anticoagulation, unfractionated heparin was administered to obtain an activated clotting time greater than 250 s within 12 h after surgery. After all the procedures, patients were on continuous aspirin 100 mg/day for their lifetime and clopidogrel 75 mg/day for 12 months.

### Off-pump CABG

All patients underwent surgery with median thoracotomy. Patients who were assisted by cardiopulmonary bypass were not included in this study. Patients with LIMA abandonment were also excluded. The anticoagulation strategy after CABG was aspirin 100 mg/day for lifetime.

### Study endpoints

The primary endpoint during follow-up was the incidence of major adverse cardiac or cerebrovascular events (MACCEs), including all-cause mortality, myocardial infarction, repeated revascularization, and stroke. In addition, cardiac death was also assessed. Cardiac death was defined as the following by the Academic Research Consortium (ARC) (11): any death related to a cardiac reason, unwitnessed mortality or an unknown cause of mortality. The secondary endpoints were in-hospital outcomes, including death, repeated revascularization, stroke, blood transfusion, reoperation for bleeding, renal failure, intra-aortic balloon pump (IABP) implantation, operation time and postoperative length of stay. If a patient experienced the same clinical event more than once, only the ﬁrst event was counted. Patients lost to follow-up were considered to have experienced no events.

### Data collection and follow-up

The clinical variables were collected from electronic medical records from hospital databases. The data collection was managed by experienced raters who were trained beforehand to ensure accordance.

The EuroSCORE II was calculated according to the literature (12). The baseline SYNTAX score can be calculated from the preprocedural angiogram using the SYNTAX score algorithm (13). The residual SYNTAX score was used to measure the completeness of revascularization (14). From the postprocedural angiogram, each coronary lesion producing ≥50% diameter stenosis in vessels ≥1.5 mm by visual estimation but left untreated was scored separately, and individual scores were added to provide the residual SYNTAX score. The residual SYNTAX score after CABG was the baseline SYNTAX score of the “native” coronary vessels, with points deducted based on the segment weighting (Leaman score) of the bypassed coronary artery (15). All the angiograms enrolled were reviewed by a dedicated interventional cardiologist who was blinded to the study design.

A predesigned chart including all of the follow-up items was applied. A phone call was the preferred follow-up method. For patients who had records of rehospitalization at the Beijing Anzhen Hospital, noteworthy information was also obtained from the hospital information system.

### Statistical analysis

Continuous variables are presented as the means ± SDs (normal distribution) or medians with interquartile ranges (skewed distribution). Comparisons were performed using Student's *t* test or the Mann‒Whitney *U* test, where appropriate. The categorical data are presented as numbers and percentages and were analysed using the chi-square test or Fisher's exact test.

We performed a propensity score-matched analysis. First, a propensity score was calculated utilizing a logistic regression model, which included all of the variables listed in Table 1. The pairs were then matched at a 1:1 ratio by utilizing a nearest-neighbour matching method (calliper value = 0.02). We assessed the balance of the variables through absolute standardized differences (ASDs). ASDs <10.0% revealed a relatively small imbalance (Supplementary Figure S1).

**Table 1:** Clinical baseline characteristics.

	Unadjusted			After propensity score matching		
	Generalized HCR (*n* = 122)	OPCABG (*n* = 379)	*P* Value	Generalized HCR (*n* = 111)	OPCABG (*n* = 111)	*P* value
Clinical characteristics						
Age (yrs)	63.1 ± 8.8	62.3 ± 8.0	0.354	63.0 ± 8.9	63.1 ± 7.7	0.910
Male	87 (71.3)	280 (73.9)	0.577	79 (71.2)	78 (70.3)	0.883
BMI (kg/m^2^)	25.1 (23.7–27.0)	25.7 (24.5–27.7)	0.028	25.2 (23.6–27.3)	25.1 (24.3–26.9)	0.757
Smoker	57 (46.7)	186 (49.1)	0.651	50 (45.0)	52 (46.8)	0.788
Hypertension	81 (66.4)	223 (58.8)	0.137	70 (63.1)	71 (64.0)	0.889
Diabetes mellitus	58 (47.5)	163 (43.0)	0.380	52 (46.8)	49 (44.1)	0.686
Hypercholesterolemia	70 (57.4)	211 (55.7)	0.741	64 (57.7)	66 (59.5)	0.785
Renal dysfunction	8 (6.6)	11 (2.9)	0.066	7 (6.3)	6 (5.4)	0.775
Previous stroke	17 (13.9)	41 (10.8)	0.349	17 (15.3)	17 (15.3)	1.000
Previous MI	19 (15.6)	111 (29.3)	0.003	18 (16.2)	17 (15.3)	0.854
Previous PCI	17 (13.9)	94 (24.8)	0.012	16 (14.4)	14 (12.6)	0.695
Atrial ﬁbrillation	5 (4.1)	23 (6.1)	0.410	5 (4.5)	5 (4.5)	1.000
Peripheral arterial disease	30 (24.6)	84 (22.2)	0.578	29 (26.1)	28 (25.2)	0.878
Chronic lung disease	7 (5.7)	24 (6.3)	0.813	6 (5.4)	7 (6.3)	0.775
LVEF (%)	60 (52–63)	54 (47–60)	0.001	60 (52–63)	59 (54–63)	0.651
EuroSCORE II	2.2 ± 1.3	1.8 ± 1.1	0.001	2.1 ± 1.2	2.1 ± 1.3	0.977
SYNTAX score	28.3 ± 3.2	28.2 ± 3.8	0.676	28.4 ± 3.3	28.1 ± 3.4	0.461
Lesion location of vessels						
LM	4 (3.3)	22 (5.8)	0.274	4 (3.6)	4 (3.6)	1.000
LAD	122 (100)	379 (100)	1.000	111 (100)	111 (100)	1.000
LCX	84 (68.9)	276 (72.8)	0.396	78 (70.3)	81 (73.0)	0.655
RCA	107 (87.7)	308 (81.3)	0.101	97 (87.4)	94 (84.7)	0.561

Values are *n* (%), mean ± SD or median with interquartile range.

HCR, hybrid coronary revascularization; OPCABG, off-pump coronary artery bypass grafting; BMI, body mass index; MI, myocardial infarction; PCI, percutaneous transluminal coronary intervention; LVEF, left ventricular ejection fraction; LM, left main coronary artery; LAD, left anterior descending coronary artery; LCX, left circumflex artery; RCA, right coronary artery.

Survival curves using the Kaplan‒Meier method were generated for the endpoints. A univariate Cox proportional hazard regression model was used to calculate the hazard ratios (HRs). Subgroup analysis was performed between the following subgroups: nonclassical HCR vs. OPCABG and nonclassical HCR vs. traditional HCR. The clinical endpoints were also reanalyzed with the Kaplan‒Meier method.

Statistical analyses were performed using SPSS 26.0 (SPSS Inc., Chicago, Illinois, USA) and Stata 15.0 (Stata, College Station, TX, USA). A two-tailed *P* value ≤0.05 was considered to indicate statistical significance.

## Results

### Patient and surgery characteristics

A total of 122 patients underwent generalized HCR, while 379 patients underwent traditional off-pump CABG with LIMA-LAD grafting from January 2019 to August 2023. After propensity score matching, 222 patients (111 in each of the 2 groups) were finally enrolled in the study. Table 1 lists the baseline characteristics before and after propensity score matching. In the matched arms, the baseline characteristics were similar between the two groups.

The surgical procedural characteristics are presented in Table 2. No statistically signiﬁcant difference was detected in the total number of anastomoses plus stents per patient between the two groups (3.2 ± 0.8 for generalized HCR vs. 2.9 ± 0.7 for OPCABG; *P* = 0.135). The residual SYNTAX score was similar between the two groups (6.3 ± 5.5 for generalized HCR vs. 6.8 ± 5.3 for OPCABG; *P* = 0.486), which indicated that they had equal completeness of revascularization.

**Table 2:** Procedural characteristics of patients in two groups.

	Generalized HCR (*n* = 111)	OPCABG (*n* = 111)	*P* value
Bypassed LIMA–LAD	111	111	1.000
Bypassed of saphenous veins	79	207	N/A
DIAG	14 (17.7)	27 (13.0)	0.346
RI	9 (11.4)	13 (6.3)	0.212
LCX	10 (12.7)	17 (8.2)	0.262
OM	25 (31.6)	55 (26.6)	0.461
RCA	2 (2.5)	25 (12.1)	0.012
PDA	18 (22.8)	58 (28.0)	0.454
PLA	1 (1.3)	12 (5.8)	0.122
Number of anastomoses			
1	42 (37.8)	0 (0)	0.001
2	59 (53.2)	24 (21.6)	0.001
3	10 (9.0)	78 (70.3)	0.001
4	0 (0)	9 (8.1)	0.003
Total number of anastomoses	190	318	N/A
Total number of stents	164	N/A	N/A
LM	2	N/A	N/A
DIAG	6	N/A	N/A
LCX	45	N/A	N/A
OM	17	N/A	N/A
RCA	73	N/A	N/A
PDA	8	N/A	N/A
PLA	13	N/A	N/A
Completeness of revascularization			
Total number of anastomoses plus stents per patient	3.2 ± 0.8	2.9 ± 0.7	0.135
Residual SYNTAX score	6.3 ± 5.5	6.8 ± 5.3	0.486

Values are *n* (%), mean ± SD.

HCR, hybrid coronary revascularization; OPCABG, off-pump coronary artery bypass grafting; LIMA, left internal mammary artery; LAD, left anterior descending coronary artery; DIAG, diagonal artery; RI, ramus intermedius artery; LCX, left circumflex artery; OM, obtuse marginal artery; RCA, right coronary artery; PDA, posterior descending artery; PLA, posterior left ventricular artery; LM, left main coronary artery.

### In-hospital outcomes

The outcomes during hospitalization are presented in Table 3. Compared with OPCABG, generalized HCR was associated with a signiﬁcantly lower IABP implantation rate (2.7% vs. 9.9%; *P* = 0.027) and shorter postoperative length of stay (6.3 ± 3.2 d vs. 7.7 ± 3.0 d; *P* = 0.001). However, no statistically signiﬁcant differences were found in in-hospital death (0 vs. 1.8%; *P* = 0.155), repeated revascularization (0.9% vs. 2.7%; *P* = 0.313), stroke (0.9% vs. 0; *P* = 0.316), blood transfusion (10.8% vs. 13.5%; *P* = 0.538), reoperation for bleeding (5.4% vs. 5.4%; *P* = 1), renal failure (0.9% vs. 1.8%; *P* = 0.561), or operation time [3.9 h [IQR, 3.5–4.3 h] vs. 4 h [IQR, 3.8–4.4 h]; *P* = 0.366] between the generalized HCR group and OPCABG group.

**Table 3:** In-hospital outcomes generalized HCR vs. OPCABG.

	Generalized HCR (*n* = 111)	OPCABG (*n* = 111)	*P* value
Death	0 (0)	2 (1.8)	0.155
Repeated revascularization	1 (0.9)	3 (2.7)	0.313
Stroke	1 (0.9)	0 (0)	0.316
Blood transfusion	12 (10.8)	15 (13.5)	0.538
Reoperation for bleeding	6 (5.4)	6 (5.4)	1.000
Renal failure	1 (0.9)	2 (1.8)	0.561
IABP implantation	3 (2.7)	11 (9.9)	0.027
Operation time (h)	3.9 (3.5–4.3)	4.0 (3.8–4.4)	0.366
Postoperative length of stay (d)	6.3 ± 3.2	7.7 ± 3.0	0.001

Values are *n* (%), mean ± SD or median with interquartile range.

HCR, hybrid coronary revascularization; OPCABG, off-pump coronary artery bypass grafting; IABP, intra-aortic balloon pump.

### Midterm follow-up outcomes

**Figure 2:**
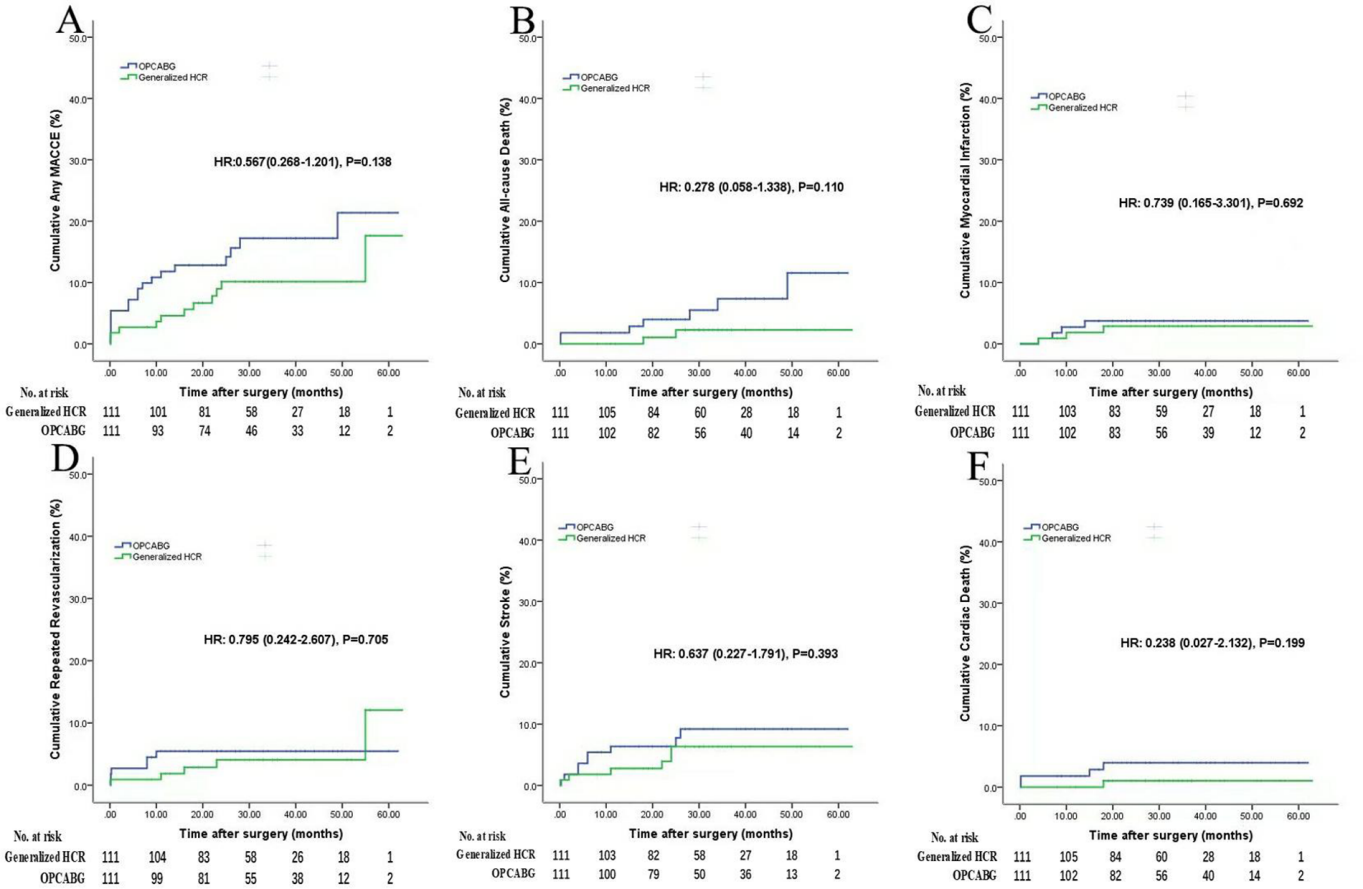
Follow-up outcomes (generalized HCR vs. OPCABG). Figures show the cumulative rates of **(A)** MACCE, **(B)** all-cause death, **(C)** myocardial infarction, **(D)** repeated revascularization, **(E)** stroke, **(F)** cardiac death. HCR, hybrid coronary revascularization; OPCABG, off-pump coronary artery bypass grafting; MACCE, major adverse cardiac or cerebrovascular event; HR, hazard ratio.

By April 2024, 100% of the generalized HCR group and 99.1% of the OPCABG group completed the information collection work, with a median follow-up time of 30.1 months [29 months (IQR, 18–42 months)]. One patient in the OPCABG group was lost to follow-up. The generalized HCR group showed a trend toward lower cumulative MACCE rates than did the OPCABG group (9.9% vs. 16.2%; HR, 0.567; 95% CL, 0.268–1.201; *P* = 0.138), but the differences were not statistically significant. In addition, no signiﬁcant difference was detected in the cumulative rates of all-cause death (1.8% vs. 6.3%; HR, 0.278; 95% CL, 0.058–1.338; *P* = 0.110), myocardial infarction (2.7% vs. 3.6%; HR, 0.739; 95% CL, 0.165–3.301; *P* = 0.692), repeated revascularization (4.5% vs. 5.4%; HR, 0.795; 95% CL, 0.242–2.607; *P* = 0.705) and stroke (5.4% vs. 8.1%; HR, 0.637; 95% CL, 0.227–1.791; *P* = 0.393) for the generalized HCR and OPCABG groups, respectively. Besides, no statistically signiﬁcant difference was found in cardiac death (0.9% vs. 3.6%; HR, 0.238; 95% CL, 0.027–2.132; *P* = 0.199) between the generalized HCR group and OPCABG group (Table 4 and Figure 2).

**Table 4:** Follow-up outcomes generalized HCR vs. OPCABG.

	Generalized HCR (*n* = 111)	OPCABG (*n* = 111)	HR (95% CL)	*P* value
Any MACCE	11 (9.9)	18 (16.2)	0.567 (0.268–1.201)	0.138
All-cause death	2 (1.8)	7 (6.3)	0.278 (0.058–1.338)	0.110
Myocardial infarction	3 (2.7)	4 (3.6)	0.739 (0.165–3.301)	0.692
Repeated revascularization	5 (4.5)	6 (5.4)	0.795 (0.242–2.607)	0.705
Stroke	6 (5.4)	9 (8.1)	0.637 (0.227–1.791)	0.393
Cardiac death	1 (0.9)	4 (3.6)	0.238 (0.027–2.132)	0.199

Values are *n* (cumulative event rate %).

HCR, hybrid coronary revascularization; OPCAB, off-pump coronary artery bypass grafting; HR, hazard ratio; CL, conﬁdence limits; MACCE, major adverse cardiac and cerebrovascular event.

### Subgroup analysis

To verify the clinical effect of this nonclassical hybridization method (PCI plus MICS-CABG), we performed subgroup analysis: nonclassical HCR (*n* = 69) vs. OPCABG (*n* = 111) and nonclassical HCR (*n* = 69) vs. traditional HCR (*n* = 42). The baseline characteristics of the subgroups are listed in Supplementary Tables S1, S2. The clinical results are summarized as follows.

**Figure 3:**
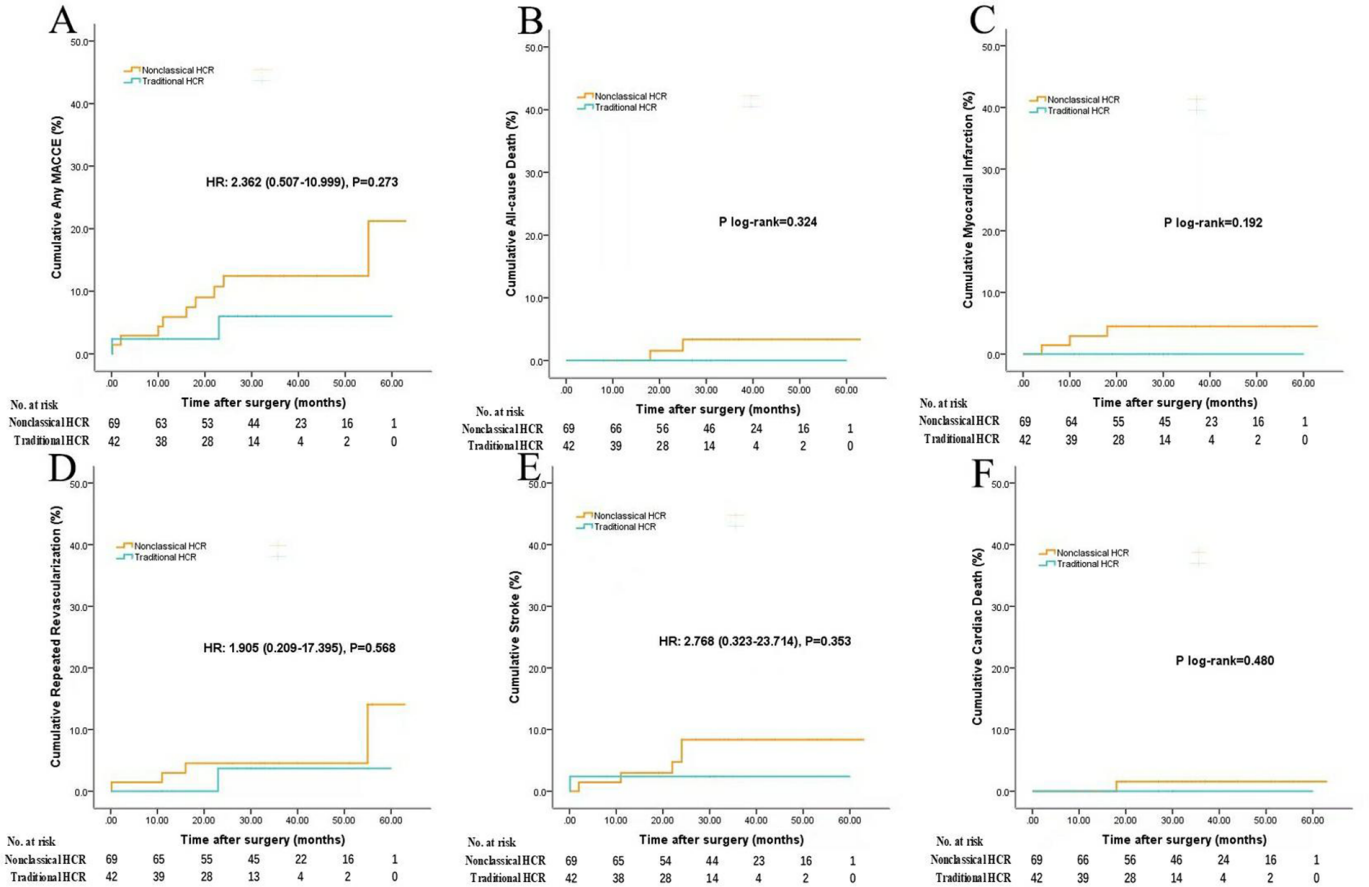
Follow-up outcomes (nonclassical HCR vs. OPCABG). Figures show the cumulative rates of **(A)** MACCE, **(B)** all-cause death, **(C)** myocardial infarction, **(D)** repeated revascularization, **(E)** stroke, **(F)** cardiac death. Abbreviations as in Figure 2.

Nonclassical HCR vs. OPCABG (Supplementary Table S3 and Figure 3): Nonclassical HCR was associated with a signiﬁcantly lower IABP implantation rate (1.4% vs. 9.9%; *P* = 0.027) and shorter postoperative length of stay (6.3 ± 3.8 d vs. 7.7 ± 3.0 d; *P* = 0.010) than OPCABG. No signiﬁcant differences were found in in-hospital death (0 vs. 1.8%; *P* = 0.262), repeated revascularization (1.4% vs. 2.7%; *P* = 0.579), stroke (0 vs. 0; *P* = 1), blood transfusion (13.0% vs. 13.5%; *P* = 0.928), reoperation for bleeding (4.3% vs. 5.4%; *P* = 0.752), renal failure (1.4% vs. 1.8%; *P* = 0.857) or operation time [4.1 h [IQR, 3.8–4.4 h] vs. 4.0 h [IQR, 3.8–4.4 h]; *P* = 0.789] between the nonclassical HCR group and OPCABG group. The results of nonclassical HCR were similar to those of OPCABG in terms of midterm follow-up outcomes.

**Figure 4:**
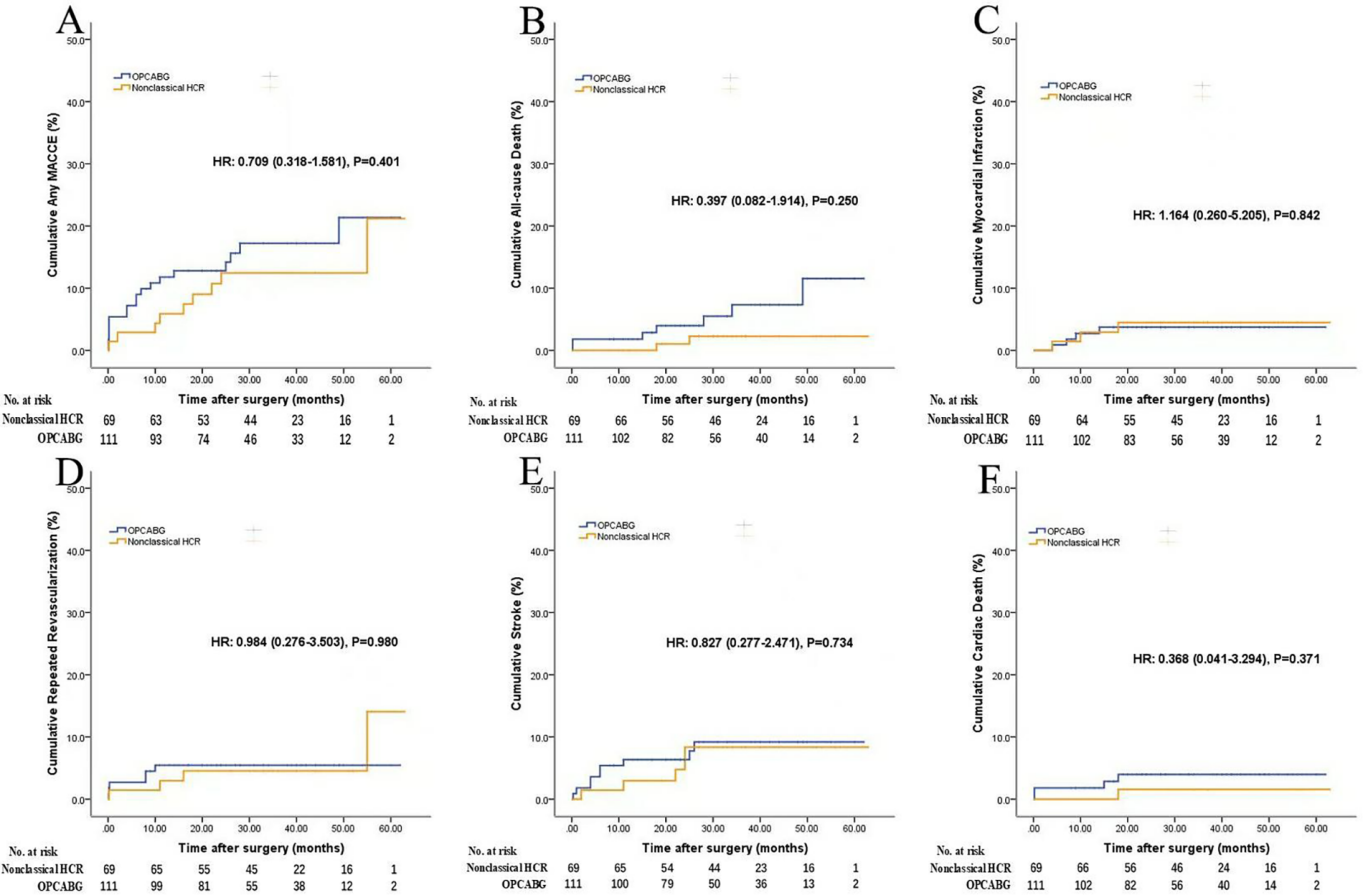
Follow-up outcomes (nonclassical HCR vs. traditional HCR). Figures show the cumulative rates of **(A)** MACCE, **(B)** all-cause death, **(C)** myocardial infarction, **(D)** repeated revascularization, **(E)** stroke, **(F)** cardiac death. Abbreviations as in Figure 2. When HR is not applicable, comparisons were performed by log-rank test.

Nonclassical HCR vs. traditional HCR (Supplementary Table S4 and Figure 4): Nonclassical HCR was associated with a longer operation time [4.1 h [IQR, 3.8–4.4 h] vs. 3.6 h [IQR, 3.3–4.1 h]; *P* = 0.043] than traditional HCR. No signiﬁcant differences were found in other in-hospital outcomes. Compared with traditional HCR, nonclassical HCR had similar results in terms of midterm follow-up outcomes.

## Discussion

This study specifically focused on the clinical effect of a generalized hybrid revascularization strategy. We found that the generalized HCR had comparable midterm outcomes in terms of total MACCE, all-cause death, myocardial infarction, repeated revascularization, stroke, and cardiac death. In-hospital outcomes showed a lower IABP implantation rate and shorter postoperative length of stay in the generalized HCR group than in the OPCABG group. No differences were found in in-hospital death, stroke, repeated revascularization, blood transfusion, reoperation for bleeding, renal failure or operation time between two groups. Compared with OPCABG and traditional HCR, nonclassical HCR showed approximately equal clinical performance for both in-hospital and follow-up outcomes.

Cardiac surgeons have been committed to enriching the idea of HCR. Bonaros N et al. (16) proposed a terminology of “advanced” HCR which deﬁned as combination of single or multiple bypass grafting AND single or multiple percutaneous interventions. In their study advanced HCR got comparable results with conventional HCR in terms of peri-operative and mid-term results. In our study generalized HCR represents a more flexible revascularization strategy involving PCI, MIDCAB or MICS-CABG. This hybridization strategy has the following advantages. First, it has broadened the indications for HCR surgery. More patients can benefit from HCR, which combines the durability and survival advantage of LIMA-LAD grafts with less invasive injury. Second, in the PCI procedure, without LAD protection, more pressure is placed on interventional cardiologists to manage complex lesions. By this HCR method, surgeons can share part of the pressure with performing anastomosis to complex non-LAD lesions in the following MICS-CABG procedure, which makes interventional cardiologists more comfortable and less resistant to cooperating with surgeons. Moreover, the safety of interventional procedures has improved. In summary, generalized HCR represents an individualized revascularization strategy that is based on the characteristics of coronary lesions.

In terms of repeated revascularization events, our ﬁndings were consistent with several recent studies (8, 17, 18). One of the representative studies in this ﬁeld was the HYBRID trial (8). In that prospective investigation, the incidence of repeat revascularization events in the HCR and CABG groups was not significantly different. However, a retrospective study from Ding et al. with a large sample size reported a greater rate of repeat revascularization in patients who underwent HCR than in those who underwent CABG (28.5% vs. 19.7%; *P* < 0.001) (19). The controversy centres on repeated revascularization events. However, a greater residual SYNTAX score (RSS) was calculated in the HCR group than in the CABG group in Ding's study (11.1 ± 6.1 vs. 6.9 ± 7.2; *P* < 0.001). A higher RSS has been demonstrated to be associated with worse outcomes (20). There is a major concern that the elevated rate of repeat revascularization is associated with incomplete revascularization. In our study, benefit from reverse HCR, PCI treatment failure could be remedied by MICS-CABG surgery. As a result, we achieved relatively satisfactory revascularization completeness. The RSS was similar between the two groups in the current study (6.3 ± 5.5 for HCR vs. 6.8 ± 5.3 for CABG; *P* = 0.486). Due to the absence of confounding factors of the completeness of revascularization, our results are more convincing.

In terms of cerebrovascular events, one of the unique advantages of the hybrid procedure is avoiding aortic clamping and manipulation, which has been demonstrated to be a predictor of postoperative cerebral infarction (21). Furthermore, studies have reported that avoiding aortic manipulation can improve neurologic outcomes (22). Previous studies have shown no signiﬁcant difference in the stroke rate between HCR and CABG, which is consistent with our study (8, 19, 23, 24). However, in the current study, there was a large concern that the use of MICS-CABG for ascending aorta anastomosis potentially increased the risk of cerebral infarction. Subgroup analyses were performed to test this concern. Compared with the CABG group and traditional HCR group, the nonclassical HCR group showed equal stroke rates for both in-hospital and follow-up outcomes. Certainly, such results may be constrained by factors such as the small sample size and short follow-up time. In clinical practice, patients who are prepared for surgery are required to undergo routine aortic CT-Angiograph. Patients with severe ascending aorta atherosclerosis should avoid aortic manipulation. Halbersma et al. (25) reported four-years outcome of OPCAB no-touch with total arterial Y-graft showing that there was a clear trend towards a reduction in stroke rate in the no-touch group. Several surgeons have performed saphenous proximal anastomosis to the LIMA (T-Graft) and achieved comparable clinical effects (26). Besides, the use of anastomosis support devices (Enclose II and Heartstring) allows proximal aortic anastomoses to be performed without a side-clamp, and significantly reduces cerebral microembolism (27). In our study, SVGs were used in all MICS-CABG patients primarily because of the flow requirements of multivessel lesions. In our experience, the internal mammary arteries in Asians are probably mostly slender. We tried T/Y-grafts anastomosed to the LIMA, but got poor blood flow. Of course, this might be related to the learning curve of this technique for our surgeons. As far as we concerned, no-touch techniques and anastomosis support devices may be alternative choices for patients with severe ascending aorta atherosclerosis.

In terms of in-hospital outcomes, apart from IABP implantation and postoperative length of stay, there was no signiﬁcant difference in other postoperative outcomes in the current study. In contrast, most previous studies have shown lower transfusion requirements for HCR than for CABG (19, 28, 29). We attribute this difference to our MICS-CABG procedure. Because of the need for exposure of the ascending aortic anastomosis and distal anastomosis of the saphenous, in most cases, a longer surgical incision is needed. In addition, traction of the chest wall during exposure may lead to intercostal artery injury and bleeding. Therefore, sufficient attention should be given to surgical haemostasis. As for operation time, an increase number of anastomoses is bound to prolong the overall duration of surgery. In subgroup analysis MICS-CABG showed signiﬁcant longer operation time than MIDCAB. IABP implantation rates was lower in generalized HCR group. It was proved the safety and reliability of this approach. Postoperative length of stay was obviously shortened in generalized HCR group, which was proved that maintaining the thoracic integrity contributes to the postoperative quick recovery.

Our study confirmed the safety and efficacy of generalized HCR. The proposed concept of generalized hybridization originated from individualized requirements of different vascular lesion characteristics. A well-designed randomized clinical trial is needed.

## Study limitations

First, this was a retrospective study from a single centre, with a relatively small sample size and short follow-up time, which may weaken the strength of our statistical analyses. Second, the OPCABG patients in the control group were treated during the same period by the same surgeons to reduce confounding bias. However, the sample size (*n* = 379) was relatively small. After propensity score matching, data loss in the HCR group was inevitable, which might have affected the statistical results. Besides, residual differences between the two groups may not be entirely mitigated by propensity score matching, leaving room for potential bias. Third, dual antiplatelet therapy during the period between PCI and MICS-CABG might have played a role in the outcomes (bleeding, stroke). In the future, we will use this generalized HCR method in simultaneous HCR procedures.

## Conclusions

The generalized HCR procedure appears to be safe and efﬁcacious, with outcomes similar to those of standard off-pump CABG and satisfactory completeness of revascularization; it represents a more flexible and individualized revascularization approach for a larger group of patients with multivessel coronary artery disease.

## Data Availability

The raw data supporting the conclusions of this article will be made available by the authors, without undue reservation.
