# Supplementary material for: Generalized hybrid coronary revascularization vs. conventional off-pump coronary artery bypass grafting for multivessel coronary artery disease
Source: Front Cardiovasc Med. 2025 Feb 21;12:1459072. doi: 10.3389/fcvm.2025.1459072 (PMC11885308; doi:10.3389/fcvm.2025.1459072)
Supplement: Supplementary file 1 [file Datasheet1.docx]

**Figure S1. Absolute Standard Difference before and after Propensity score matching**

**
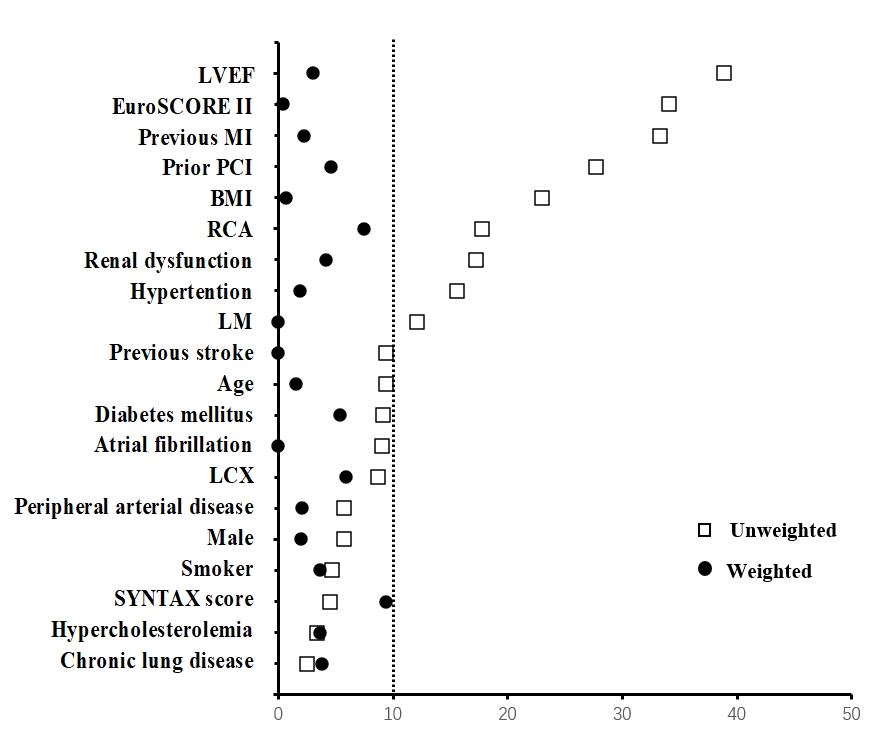
**

**Tables**

| **Table S1 Baseline Characteristics Nonclassical HCR versus OPCABG** | | | |
| --- | --- | --- | --- |
|  | **Nonclassical HCR**  **（n=69）** | **OPCABG**  **(n=111)** | **P Value** |
| **Clinical Characteristics** | | | |
| Age (yrs) | 62.3±9.1 | 63.1±7.7 | 0.530 |
| Male | 51(73.9) | 78(70.3) | 0.598 |
| BMI (kg/m²) | 25.2(23.8-27.4) | 25.1(24.3-26.9) | 0.549 |
| Smoker | 34(49.3) | 52(46.8) | 0.751 |
| Hypertension | 45(65.2) | 71(64.0) | 0.864 |
| Diabetes mellitus | 34(49.3) | 49(44.1) | 0.502 |
| Hypercholesterolemia | 38(55.1) | 66(59.5) | 0.562 |
| Renal dysfunction | 6(8.7) | 6(5.4) | 0.390 |
| Previous stroke | 13(18.8) | 17(15.3) | 0.537 |
| Previous MI | 12(17.4) | 17(15.3) | 0.713 |
| Previous PCI | 10(14.5) | 14(12.6) | 0.718 |
| Atrial ﬁbrillation | 1(1.4) | 5(4.5) | 0.267 |
| Peripheral arterial disease | 21(30.4) | 28(25.2) | 0.445 |
| Chronic lung disease | 5(7.2) | 7(6.3) | 0.806 |
| LVEF (%) | 60(52-64) | 59(54-63) | 0.798 |
| EuroSCORE II | 2.2±1.2 | 2.1±1.3 | 0.557 |
| SYNTAX score | 28.4±3.0 | 28.1±3.4 | 0.542 |
| **Lesion location of vessels** | | | |
| LM | 2(2.9) | 4(3.6) | 0.798 |
| LAD | 69(100) | 111(100) | 1.000 |
| LCX | 62(89.9) | 81(73.0) | 0.006 |
| RCA | 64(92.8) | 94(84.7) | 0.108 |
| Values are n (%), mean±SD or median with interquartile range.  Abbreviations as in Table 1. | | | |

| **Table S2 Baseline Characteristics Nonclassical HCR versus Traditional HCR** | | | |
| --- | --- | --- | --- |
|  | **Nonclassical HCR**  **（n=69）** | **Traditional HCR (n=42)** | **P Value** |
| **Clinical Characteristics** | | | |
| Age (yrs) | 62.3±9.1 | 64.1±8.7 | 0.313 |
| Male | 51(73.9) | 28(66.7) | 0.414 |
| BMI (kg/m²) | 25.2(23.8-27.4) | 25.2(23.3-26.9) | 0.438 |
| Smoker | 34(49.3) | 16(38.1) | 0.251 |
| Hypertension | 45(65.2) | 25(59.5) | 0.547 |
| Diabetes mellitus | 34(49.3) | 18(42.9) | 0.511 |
| Hypercholesterolemia | 38(55.1) | 26(61.9) | 0.480 |
| Renal dysfunction | 6(8.7) | 1(2.4) | 0.184 |
| Previous stroke | 13(18.8) | 4(9.5) | 0.186 |
| Previous MI | 12(17.4) | 6(14.3) | 0.667 |
| Previous PCI | 10(14.5) | 6(14.3) | 0.976 |
| Atrial ﬁbrillation | 1(1.4) | 4(9.5) | 0.047 |
| Peripheral arterial disease | 21(30.4) | 8(19.0) | 0.185 |
| Chronic lung disease | 5(7.2) | 1(2.4) | 0.272 |
| LVEF (%) | 60(52-64) | 58(52-63) | 0.138 |
| EuroSCORE II | 2.2±1.2 | 1.9±1.2 | 0.213 |
| SYNTAX score | 28.4±3.0 | 28.4±3.8 | 0.913 |
| **Lesion location of vessels** | | | |
| LM | 2(2.9) | 2(4.8) | 0.609 |
| LAD | 69(100) | 42(100) | 1.000 |
| LCX | 62(89.9) | 16(38.1) | 0.001 |
| RCA | 64(92.8) | 33(78.6) | 0.029 |
| Values are n (%), mean±SD or median with interquartile range.  Abbreviations as in Table 1. | | | |

| **Table S3 Clinical Outcomes Nonclassical** **HCR versus OPCABG** | | | | |
| --- | --- | --- | --- | --- |
|  | **Nonclassical HCR**  **(n=69)** | **OPCABG**  **(n=111)** | **HR (95% CL)** | **P Value** |
| **In-hospital Outcomes** |  |  |  |  |
| Death | 0(0) | 2(1.8) | N/A | 0.262 |
| Repeated revascularization | 1(1.4) | 3(2.7) | N/A | 0.579 |
| Stroke | 0(0) | 0(0) | N/A | 1.000 |
| Blood transfusion | 9(13.0) | 15(13.5) | N/A | 0.928 |
| Reoperation for bleeding | 3(4.3) | 6(5.4) | N/A | 0.752 |
| Renal failure | 1(1.4) | 2(1.8) | N/A | 0.857 |
| IABP implantation | 1(1.4) | 11(9.9) | N/A | 0.027 |
| Operation time (h) | 4.1(3.8-4.4) | 4.0(3.8-4.4) | N/A | 0.789 |
| Postoperative length of stay (d) | 6.3±3.8 | 7.7±3.0 | N/A | 0.010 |
| **Follow-Up Outcomes** |  |  |  |  |
| Any MACCE | 9(13.0) | 18(16.2) | 0.709(0.318-1.581) | 0.401 |
| All-cause death | 2(2.9) | 7(6.3) | 0.397(0.082-1.914) | 0.250 |
| Myocardial infarction | 3(4.3) | 4(3.6) | 1.164(0.260-5.205) | 0.842 |
| Repeated revascularization | 4(5.8) | 6(5.4) | 0.984(0.276-3.503) | 0.980 |
| Stroke | 5(7.2) | 9(8.1) | 0.827(0.277-2.471) | 0.734 |
| Cardiac death | 1(1.4) | 4(3.6) | 0.368(0.041-3.294) | 0.371 |
| Values are n (%), mean±SD or median with interquartile range.  Abbreviations as in Table 3 plus 4. | | | | |

| **Table S4 Clinical Outcomes Nonclassical** **HCR versus Traditional HCR** | | | | |
| --- | --- | --- | --- | --- |
|  | **Nonclassical HCR**  **(n=69)** | **Traditional HCR**  **(n=42)** | **HR (95% CL)** | **P Value** |
| **In-hospital Outcomes** |  |  |  |  |
| Death | 0(0) | 0(0) | N/A | 1.000 |
| Repeated revascularization | 1(1.4) | 0(0) | N/A | 0.433 |
| Stroke | 0(0) | 1(2.4) | N/A | 0.198 |
| Blood transfusion | 9(13.0) | 3(7.1) | N/A | 0.332 |
| Reoperation for bleeding | 3(4.3) | 3(7.1) | N/A | 0.528 |
| Renal failure | 1(1.4) | 0(0) | N/A | 0.433 |
| IABP implantation | 1(1.4) | 2(4.8) | N/A | 0.297 |
| Operation time (h) | 4.1(3.8-4.4) | 3.6(3.3-4.1) | N/A | 0.043 |
| Postoperative length of stay (d) | 6.3±3.8 | 6.3±1.7 | N/A | 0.921 |
| **Follow-Up Outcomes** |  |  |  |  |
| Any MACCE | 9(13.0) | 2(4.8) | 2.362(0.507-10.999) | 0.273 |
| All-cause death | 2(2.9) | 0(0) | N/A | 0.324* |
| Myocardial infarction | 3(4.3) | 0(0) | N/A | 0.192* |
| Repeated revascularization | 4(5.8) | 1(2.4) | 1.905(0.209-17.395) | 0.568 |
| Stroke | 5(7.2) | 1(2.4) | 2.768(0.323-23.714) | 0.353 |
| Cardiac death | 1(1.4) | 0(0) | N/A | 0.480* |
| Values are n (%), mean±SD or median with interquartile range.  Abbreviations as in Table 3 plus 4. *Log-rank test | | | | |
